# Study of the association between the donors and recipients angiotensin-converting enzyme insertion/deletion gene polymorphism and the acute renal allograft rejection

**DOI:** 10.12860/jnp.2015.13

**Published:** 2015-07-01

**Authors:** Jalal Azmandian, Mohamadamir Mohamadifar, Sara Rahmanian-Koshkaki, Mohammad Mehdipoor, Mohamad-Hadi Nematollahi, Amin Saburi, Ali Mandegary

**Affiliations:** ^1^Physiology Research Center, Institute of Neuropharmacology, Kerman University of Medical Sciences, Kerman, Iran; ^2^Department of Nephrology, Urology and Renal Transplantation, Afzalipoor Hospital, University of Medical Sciences, Kerman, Iran; ^3^Pharmaceutics Research Center, Institute of Neuropharmacology, Kerman University of Medical Sciences, Kerman, Iran; ^4^Birjand Atherosclerosis and Coronary Artery Research Center, Birjand University of Medical Sciences, Birjand, Iran; ^5^Gastroenterology and Hepatology Research Center, Institute of Basic and Clinical Physiology Sciences, Kerman University of Medical Sciences, Kerman, Iran

**Keywords:** Angiotensin-converting-enzyme, Gen polymorphism, Acute rejection, Kidney transplant

## Abstract

*Background:* Angiotensin converting enzyme (ACE) is involved in various pathophysiological conditions including renal function. ACE levels are under genetic control.

*Objectives:* This study was designed to investigate the association between the donors and recipients ACE-I/D gene polymorphism and risk of acute rejection outcome in renal allograft recipients.

*Patients and Methods:* ACE-I/D polymorphism was determined in 200 donor-recipient pairs who had been referred to Afzalipour hospital in Kerman. ACE-I/D polymorphism was detected using polymerase chain reaction (PCR). Acute rejection (AR) during at least six months post-transplantation was defined as a 20% increase in creatinine level from the postoperative baseline in the absence of other causes of graft dysfunction which responded to antirejection therapy.

*Results:* The observed allele frequencies were II 9.8%, ID 35.6% and DD 44.4% in donors and II 9.8%, ID 35.1% and DD 52.7% in recipients. There were no significant association between ACE genotypes and AR episodes (OR_ID_=0.96 [0.18-5.00] and OR_DD_: 1.24 [0.25-6.07] for the donors) and (OR_ID_: 0.29 [0.06-1.45] and OR_DD_: 0.75 [0.19-2.90] for the recipients).

*Conclusions:* It seems that donor and recipient ACE-I/D genotype might not be a risk factor for acute renal allograft rejection. However, due to conflicting results from this and other studies, multicenter collaborative studies with more participants and concomitant evaluation of ACE polymorphism with other polymorphisms in renin–angiotensin system (RAS) are suggested to determine whether ACE genotypes are significant predictors of renal allograft rejection.

**Implication for health policy/practice/research/medical education:**

In a study on 200 donor-recipient pairs, we found that donor and recipient ACE-DD genotype might not be a risk factor for acute renal allograft rejection.

## 1. Background

Kidney transplant is the last therapy line and elective treatment for patients with advanced and chronic renal disorders, respectively, and improve survival and quality of these patients’ life-time (1,2). Indeed, the most common transplanted organ is related to kidney. Surgical procedure of kidney transplant is simple, and in the point of technically is more convenient than heart and liver transplant. In the last 2 decades, the short-term outcomes of transplant have improved significantly. However, improving condition has not seen for long-term consequences (3,4). Chronic allograft dysfunction (CAD) of transplanted kidney is a general term used to describe different periods. Hyper-acute, acute, and chronic rejection reactions occur up to 24 hours, the first few weeks, and a few months to several years after transplantation, respectively. Immunological incompatibilities are the most important factor involved in transplanted kidney outcome (5). The key reason for allograft kidney loss is chronic attrition. It is not fully known factors, leading to such disorders, however, these factors might include genetic predisposition and underlying renal diseases. In a series of investigations, we have detected the association of some genetic polymorphisms with acute rejection (AR) and delayed graft function in the renal transplanted patients the Iranian patients (6-8). Recently, several studies have been reported that effective genets involving in the regulation of blood pressure, endothelial cells and inflammation response play dramatic role in this disease pathogenesis. It is considered genes determining renin-angiotensin II are independent factors, effecting on transplanted kidney function (9,10). Renin-angiotensin system (RAS) is one of the important mediators involved in physiology of cardiovascular and kidney system, and have the substantial role in etiology of cardiovascular system (11). Recent studies have been demonstrated a well-known contribution between mentioned system and development of several diseases, including diabetes mellitus and chronic allograft nephropathy (12). Moreover, the excessive activity of this system is related to short-term life and survival of kidney transplant (13,14). The important of these studies and evidences were substantial increased, following the determination of the role of RAS as an independent factor for progression of renal attrition and end-stage renal disease (ESRD) cause (15). Hence, polymorphism of genes regulating renin-angiotensin might be the most important mediators of transplanted kidney outcome, and are important in determining the prognosis of renal allograft (13). Some genotypes of renin-angiotensin polymorphism including, angiotensin gene (A1166C), angiotensin-converting-enzyme gene, and angiotensin receptor type (M235T), regulating mentioned system associated to high activity of this system, and decreased long-term outcome of renal transplant (16,17). More than 100 polymorphisms in angiotensin converting enzyme (ACE) gene have been reported. One of the most considered polymorphism in clinical study is ACE gene identified for the first time by Rigat and colleagues in 1990 (18). ACE polymorphism is based on being or lake a 287 bp known Alu in intron 16 from chromosome 17. Polymorphism has been shown as being I (addition; insertion), or lack of D (Deletion; deleted sequence) in 287 bp fragment, and would determine the plasma enzyme levels. In Caucasians, I allele and D allele associated with lower and higher ACE activity in bloodstream, respectively, explaining about half of the variation in circulating ACE level through I/D polymorphism (18). D allele of ACE gene leads to high level of this enzyme in plasma (19). It has been demonstrated that D allele (Deletion; deleted sequence) was related to increased serum level of ACE enzyme, however, I allele (addition; insertion) had the opposite role, and individuals with I/I genotype is developed less to diabetic nephropathy (20,21). It has been reported that D/D genotype would enhance the risk of allograft rejection compared to other genotypes. However, there are controversial results about effect of ACE gene polymorphism on development of diabetic nephropathy (22,23).

Among different polymorphism of RAS, angiotensinogen polymorphism with M and T alleles as the initial composition of the system, and angiotensin-converting-enzyme as the key enzyme to convert angiotensin I to II with D and I alleles, and type 1 angiotensin II receptor as the last and effective terminus related to this system with A and C alleles are substantial important. Studies showed that RAS activity is markedly in several genotypes, including angiotensinogen (TT, AA), angiotensin-converting enzyme (DD), type 1 angiotensin II receptor (CC) (24,25). Furthermore, clinical studies have demonstrated that AA genotypes of angiotensinogen and DD for converting enzyme associated with decreased survival of renal transplant (14,16). D allele or DD genotype is considered to dispose individual to several diseases, including Alzheimer, diabetes mellitus, polycystic kidney disease, hypertension, coronary heart disease, and risk of miscarriage (26,27).

These different in enzyme activity resulted from genotype ACE gene could effect on therapy responses to inhibitors, and is caused to interpersonal differences in cardiovascular or renal responses to these drugs (28,29). Since proteinuria and hypertension are the most important risk factors associated with dysfunction of transplanted kidney, and an increase in survival and function of renal transplanted with using ACE inhibitors was observed, thus determining the interaction of hypertension and proteinuria after renal transplant with ACE gene polymorphism is necessary.

## 2. Objectives

Considering the important role of ACE in various pathophysiological conditions including renal function, we aimed to investigate the association between I/D polymorphism of ACE gene and incident of AR event in kidney transplant recipients.

## 3. Patients and Methods

### 3.1. Subjects

This study was a prospective study. The study population included 200 donor-recipient pairs of kidney transplantation in Afzalipoor hospital. Inclusion criteria for patients were grafting transplant from a living person, the first renal transplant, and signing the consent form. This research was conducted according to the principles of Helsinki Declaration (1964), and the world physicians association in Turkey (1975). It has been explained to all voluntary recipients about research, and condition being in the study. Moreover, consent forms were taken from all patients. Every participant was identified with a special code, and recipients’ individual characteristics were only for the project manages (6-8).

### 3.2. Transplant outcomes

AR during at least 6 months of post-transplantation was defined as a 20% increase in creatinine level from the postoperative baseline in the absence of other causes of graft dysfunction that responded to antirejection therapy (27,30).

### 3.3. ACE-I/D genotyping

Peripheral bloods was taken from 200 patients before kidney transplant, and harvested in tubes containing EDTA. After isolation of leukocytes, DNA extraction was conducted from buffy coat through digestion with proteniase K and salting out. In this method, leukocytes were lysed, and DNA was sorted from proteins with using proteinase-K. DNA was precipitated with saturated salt solution, washed through alcohol. The purity and quality of DNA isolated determined by spectrophotometric method (OD260nm/OD280). The polymorphism of ACE-I/D (NG_011648.1, NCBI RefSeq Nucleic) was determined using polymerase chain reaction (PCR) as previously described by others (31,32). Briefly, a fragment of ACE gene amplified using the following primers: sense 5’-GCC CTG CAG GTG TCT GCA GCA TGT-3’ and antisense 5’-GGA TGG CTC TCC CCG CCT TGT CTC-3’. The PCR products were then analyzed by electrophoresis in 2% agarose gels and visualized by ethidium bromide staining. The PCR products yielded bands of 597 bp in I/I homozygotes, 319 bp in D/D homozygotes, and both 2 bands (319 bp + 597 bp) in heterozygotes.

### 3.4. Ethical issues

The research followed the tenets of the Declaration of Helsinki and the world physicians association in Turkey (1975). Informed consents were obtained. All patients took part in this study voluntary. The research was approved by ethical committee of Kerman University of Medical Sciences.

### 3.5. Statistical analysis

After data collection, the transplantation findings were recorded according to rejection episodes for every polymorphic genotype. The significant differences of graft rejection between groups (heterozygote, homozygote, and wild-type) were analyzed through test of independence (chi-square test). The correlation between ACE genetic polymorphism and graft function were analyzed by logistic regression. Odds ratios (OR) and 95% CI were used to estimate the risk of the association between AR and a specific genotype Moreover, *t* test and analysis of variance (ANOVA) were used to compare age and body mass index (BMI) in donor and recipient groups, and various genotypes. SPSS version 20 was used to analyzed, and *P*<0.05 was considered the significant level.

## 4. Results

**Figure 1:**
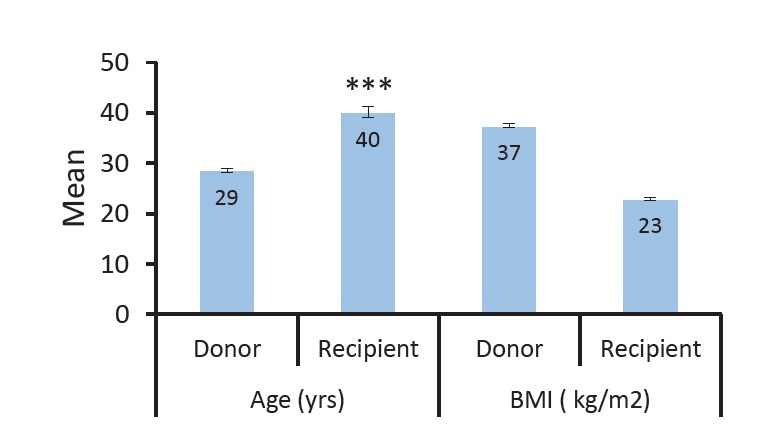
Age and BMI of the donors and recipients in this study, ***P < 0.001.

Findings indicated significant difference between the mean age of the donor and the recipient (*P *< 0.05; Figure 1). The mean age of the recipients was significantly higher than the age of the donor. Moreover, between the BMI mean of the donor and the recipients were significant difference (*P *< 0.05). The mean of BMI related to the recipients were significantly lower than BMI of donors.

### 4.1. Association of donor ACE-I/D polymorphism with AR

**Figure 2:**
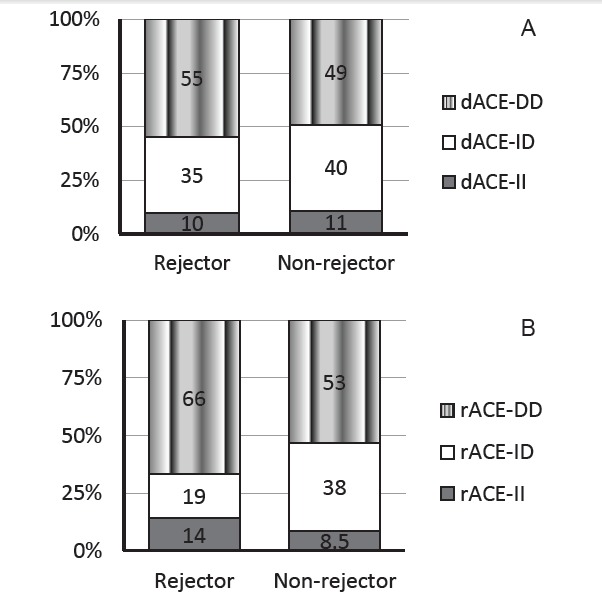
Frequency of different genotypes of the donors (A) and recipients (B) ACE-I/D in the patients with and without allograft rejection after kidney transplantation

Association between donor ACE-I/D polymorphism and the frequency of AR has been shown in the Figure 2 and Table 1. The frequencies of ID and DD alleles were not significantly different in the grafts with and without AR (OR_ID_=0.96 [0.18-5.00] and OR_DD_: 1.24 [0.25-6.07] for the donors).

**Table 1:** Association of the donors ACE-I/D polymorphism with AR in kidney transplant patients

**Genotype**	**AR No. (%)**	**No-ARNo. (%)**	***P *** **value**	**OR (CI)**
Donor ACE				
II	2 (10)	18 (11)		1
ID	7 (35)	66 (40)	0.96	0.96 (0.18-4.99)
DD	11 (55)	80 (49)	0.79	1.24 (0.25-6.07)
Total	20 (100)	164 (100)		

Abbreviations: AR‏, acute rejection; ; ACE, angiotensis converting enzyme; OR, Odds ratios.

### 4.2. Association of recipient ACE-I/D polymorphism with AR

Figure 2 and Table 2 summarize the frequency of recipient ACE-I/D alleles in the patients with and without AR. The frequencies of ID and DD alleles were not significantly different in the patients with and without AR (OR_ID_: 0.29 [0.06-1.45] and OR_DD_: 0.75 [0.19-2.90] for the recipients).

**Table 2:** Association of the recipients ACE-I/D polymorphism with AR in kidney transplant patients

**Genotype**	**AR** **No. (%)**	**No-AR** **No. (%)**	***P*** ** value**	**OR (CI)**
Recipient ACE				
II	3 (14)	15 (8.5)		1
ID	4 (19)	68 (38)	0.13	0.29 (0.06-1.45)
DD	14 (66)	94 (53)	0.67	0.75 (0.19-2.90)

Abbreviations: AR‏, acute rejection; ACE, angiotensin converting enzyme; OR, Odds ratios.

## 5. Discussion

Chronic renal dysfunction is the main cause of kidney disorder in transplant recipient. The factors involved in this disorders are not completely understood. However, it has been reported the role of underlying kidney disease and genetic and environmental factors in such pathology.

Genes regulating renin-angiotensin in transplant recipients might affect kidney function as independent factor (10). Gene polymorphism involved in different component of RAS was associated to several diseases. From the mid-1990s, considerable studies on genetic polymorphism of the renin-angiotensin have been done as part of effective agents in prediction of renal diseases (33). On the other hand, chronically poor graft function is the main factor for losing transplanted kidney. Factors leading to these disorders are not fully understood, however, combination of background renal diseases and genetic and environmental factors has been introduced as factors involved in chronic improper function of graft organ.

Based on histopathological findings, genetic factors of transplant recipient influence the progression of chronic renal dysfunction through proliferation of various endothelial cells and mesenchyme. It is considered, genes found in RAS, effecting on transplanted kidney outcome (10).

As previously mentioned, angiotensin II affects hemodynamic kidney, and induces the proliferation cells, and synthesis extracellular matrix proteins, developing the advanced fibrotic disease in various organs. Hence, polymorphisms in genes regulating renin-angiotensin activity are the most important factors, determining transplanted renal outcome. Consequently, finding genes mediating RAS is substantial important. Recent several studies have indicated that hyper activity of such system associated to decreased function and survival transplanted renal (9,13). Previous studied have been demonstrated correlation between the genotypes of ACE (I/D) and period of graft loss in high-risk recipient group. However, the results of this study indicated that ACE (I/D) polymorphism had no significant correlation with AR event in kidney transplanted patients in Afzalipour hospital in Kerman, Iran. Here, special interaction has not observed between the mean arterial pressure (MAP) and various ACE gene genotypes in recipient patients.

Due to the different and sometimes contradictory findings, difference between races, gene-gene, and gene-environment interactions, in the present study investigated the effect of ACE I/D gene polymorphism, and incident of AR in kidney transplant recipients in our hospital, in Kerman.

In addition, the age of donor and transplant recipients compared to according to ACE gene genotypes. On the base results, the mean age of transplant donor and recipient were lower in type II genotypes and type DD genotypes than other genotype groups, respectively. It seems, the racial and geographic variations cause the different interactions I/D polymorphism related to ACE genes for kidney patients. As a result, the more research is necessary in this field.

## 6. Conclusions

It seems that donor and recipient ACE-DD genotype might not be a risk factor for acute renal allograft rejection. However, due to conflicting results from this and other studies, multicenter collaborative studies with more participants and concomitant evaluation of ACE polymorphism with other polymorphisms in RAS system are suggested to determine whether ACE genotypes are significant predictors of renal allograft rejection.

## 7. Limitations of the study

The limitations of the study were no possibility for confirming the AR by biopsy and high number of genotype missing.

## Acknowledgments

We are grateful to the patients who participated in this study. The authors would like to thank Mrs. Haghparast for her assistance to collect the samples and filling the questionnaire.

## Authors’ contribution

Collecting data: MAM and ARK. Genotyping: MM and MHN. Statistical analysis: AM. Drafting manuscript: AS and AM. Study supervision: JA and AM.

## Conflicts of interest

The authors declared no competitive interests.

## Funding/Support

The study was supported by grants from deputy of research, Kerman University of Medical Sciences to AM (grant No. 92270).

## References

[1] Tonelli M, Wiebe N, Knoll G, Bello A, Browne S, Jadhav D (2011). Systematic review: kidney transplantation compared with dialysis in clinically relevant outcomes. Am J Transplant.

[2] Davis CL, Delmonico FL (2005). Living-donor kidney transplantation: a review of the current practices for the live donor. J Am Soc Nephrol.

[3] Karthikeyan V, Karpinski J, Nair RC, Knoll G (2004). The burden of chronic kidney disease in renal transplant recipients. Am J Transplant.

[4] Meier-Kriesche HU, Schold JD, Kaplan B (2004). Long-term renal allograft survival: have we made significant progress or is it time to rethink our analytic and therapeutic strategies?. Am J Transplant.

[5] Pour-Reza-Gholi F, Daneshvar S, Nafar M, Firouzan A, Farrokhi F, Einollahi B (2005). Potential risk factors for hypersensitization reflected by panel-reactive antibodies in dialysis patients. Transplant Proc.

[6] Azmandian J, Mandegary A, Saber A, Torshabi M, Etminan A, Ebadzadeh MR (2012). Chemokine receptor 2-V64I and chemokine receptor 5-Delta32 polymorphisms and clinical risk factors of delayed graft function and acute rejection in kidney transplantation. Iran J Kidney Dis.

[7] Mandegary A, Azmandian J, Soleymani S, Pootari M, Habibzadeh SD, Ebadzadeh MR (2013). Effect of donor tumor necrosis factor-alpha and interleukin-10 genotypes on delayed graft function and acute rejection in kidney transplantation. Iran J Kidney Dis.

[8] Mandegary A, Rahmanian-Koshkaki S, Mohammadifar MA, Pourgholi L, Mehdipour M, Etminan A (2015). Investigation of association between donors’ and recipients’ NADPH oxidase p22(phox) C242T polymorphism and acute rejection, delayed graft function and blood pressure in renal allograft recipients. Transpl Immunol.

[9] Wolf G, Neilson EG (1993). Angiotensin II as a renal growth factor. J Am Soc Nephrol.

[10] Slowinski T, Diehr P, Kleemann P, Fritsche L, Renders L, Budde K (2004). No association between renin-angiotensin system gene polymorphisms and early and long-term allograft dysfunction in kidney transplant recipients. Nephrol Dial Transplant.

[11] Wang JG, Staessen JA (2000). Genetic polymorphisms in the renin-angiotensin system: relevance for susceptibility to cardiovascular disease. Eur J Pharmacol.

[12] Rodriguez-Moreno A, Sanchez-Fructuoso AI, Ridao-Cano N, Calvo N, Conesa J, Gomez-Gallego F (2005). Association of the genetic polymorphisms of the renin-angiotensin system with kidney graft long-term outcome: preliminary results. Transplant Proc.

[13] Akcay A, Ozdemir FN, Atac FB, Sezer S, Verdi H, Arat Z (2004). Angiotensin-converting enzyme genotype is a predictive factor in the peak panel-reactive antibody response. Transplant Proc.

[14] Abdi R, Tran TB, Zee R, Brenner BM, Milford EL (2001). Angiotensin gene polymorphism as a determinant of posttransplantation renal dysfunction and hypertension. Transplantation.

[15] Hunley TE, Julian BA, Phillips JA 3rd, Summar ML, Yoshida H, Horn RG (1996). Angiotensin converting enzyme gene polymorphism: potential silencer motif and impact on progression in IgA nephropathy. Kidney Int.

[16] Nicod J, Richard A, Frey FJ, Ferrari P (2002). Recipient RAS gene variants and renal allograft function. Transplantation.

[17] Reich H, Duncan JA, Weinstein J, Cattran DC, Scholey JW, Miller JA (2003). Interactions between gender and the angiotensin type 1 receptor gene polymorphism. Kidney Int.

[18] Rigat B, Hubert C, Alhenc-Gelas F, Cambien F, Corvol P, Soubrier F (1990). An insertion/deletion polymorphism in the angiotensin I-converting enzyme gene accounting for half the variance of serum enzyme levels. J Clin Invest.

[19] Tiret L, Rigat B, Visvikis S, Breda C, Corvol P, Cambien F (1992). Evidence, from combined segregation and linkage analysis, that a variant of the angiotensin I-converting enzyme (ACE) gene controls plasma ACE levels. Am J Hum Genet.

[20] Arfa I, Abid A, Nouira S, Elloumi-Zghal H, Malouche D, Mannai I (2008). Lack of association between the angiotensin-converting enzyme gene (I/D) polymorphism and diabetic nephropathy in Tunisian type 2 diabetic patients. J Renin Angiotensin Aldosterone Syst.

[21] Movva S, Alluri RV, Komandur S, Vattam K, Eppa K, Mukkavali KK (2007). Relationship of angiotensin-converting enzyme gene polymorphism with nephropathy associated with Type 2 diabetes mellitus in Asian Indians. J Diabetes Complications.

[22] Azarpira N, Bagheri M, Raisjalali GA, Aghdaie MH, Behzadi S, Salahi H (2009). Angiotensinogen, angiotensine converting enzyme and plasminogen activator inhibitor-1 gene polymorphism in chronic allograft dysfunction. Mol Biol Rep.

[23] Glenn KL, Du ZQ, Eisenmann JC, Rothschild MF (2009). An alternative method for genotyping of the ACE I/D polymorphism. Mol Biol Rep.

[24] Lalouel JM, Rohrwasser A, Terreros D, Morgan T, Ward K (2001). Angiotensinogen in essential hypertension: from genetics to nephrology. J Am Soc Nephrol.

[25] Meier-Kriesche HU, Schold JD, Kaplan B (2004). Long-Term Renal Allograft Survival: Have we Made Significant Progress or is it Time to Rethink our Analytic and Therapeutic Strategies?. American Journal of Transplantation.

[26] Scharplatz M, Puhan MA, Steurer J, Bachmann LM (2004). What is the impact of the ACE gene insertion/deletion (I/D) polymorphism on the clinical effectiveness and adverse events of ACE inhibitors?--Protocol of a systematic review. BMC Med Genet.

[27] Singh R, Kapoor R, Srivastava A, Mittal RD (2009). Impact of chemokine receptor CCR2 and CCR5 gene polymorphism on allograft outcome in North Indian renal transplant recipients. Scand J Immunol.

[28] De Jong PE, de Zeeuw D (2005). Renoprotective therapy: is it blood pressure or albuminuria that matters?. Lancet.

[29] Niu T, Chen X, Xu X (2002). Angiotensin converting enzyme gene insertion/deletion polymorphism and cardiovascular disease: therapeutic implications. Drugs.

[30] Abdi R, Tran TB, Sahagun-Ruiz A, Murphy PM, Brenner BM, Milford EL (2002). Chemokine receptor polymorphism and risk of acute rejection in human renal transplantation. J Am Soc Nephrol.

[31] Tripathi G, Dharmani P, Khan F, Sharma RK, Pandirikkal V, Agrawal S (2006). High prevalence of ACE DD genotype among north Indian end stage renal disease patients. BMC Nephrol.

[32] van Suylen RJ, Wouters EF, Pennings HJ, Cheriex EC, van Pol PE, Ambergen AW (1999). The DD genotype of the angiotensin converting enzyme gene is negatively associated with right ventricular hypertrophy in male patients with chronic obstructive pulmonary disease. Am J Respir Crit Care Med.

[33] Frishberg Y, Becker-Cohen R, Halle D, Feigin E, Eisenstein B, Halevy R (1998). Genetic polymorphisms of the renin-angiotensin system and the outcome of focal segmental glomerulosclerosis in children. Kidney Int.

